# Combined DFT Protocol for the Calculation of One-Bond ^31^P-^31^P Indirect Nuclear Spin–Spin Couplings

**DOI:** 10.3390/molecules31111831

**Published:** 2026-05-26

**Authors:** Svetlana A. Kondrashova, Shamil K. Latypov

**Affiliations:** Arbuzov Institute of Organic and Physical Chemistry, FRC Kazan Scientific Center of RAS, Kazan 420088, Tatarstan, Russia; kondrashovamail@gmail.com

**Keywords:** NMR parameters, indirect nuclear spin–spin couplings, density functional theory, organophosphorus compounds

## Abstract

The comparative analysis of calculated and experimental one-bond ^31^P-^31^P indirect nuclear spin–spin couplings for a wide range of structures, including P-P bonds, has shown that, on the whole, it is possible to estimate ^1^*J*_PP_ fairly accurately using even modest levels of theory. However, in order to reduce systematic errors, it is necessary to carry out a linear correction procedure specific to different groups of compounds. Certain difficulties may arise only for diphosphanes (R_1_R_2_P–PR_1_R_2_) that are in solution in fast (in NMR time scale) exchange of conformers with close populations. In practice, a relatively simple PBE0/6-31G(d)//PBE0/6-31G(d) combination is sufficient for calculating the ^1^*J*_PP_ with practically reliable accuracy. The efficiency of the proposed protocol is demonstrated using the example of more subtle structural features—the isomeric structure. The proposed approach allowed for the absolute sign of ^1^*J*_PP_ in a number of cases where it is unknown experimentally.

## 1. Introduction

One of the important steps in the structure-property correlation is the establishment of fine structural details involving conformational, isomeric, and tautomeric equilibria. Such information is especially important in biochemical studies [1,2,3], as well as in the development of new catalysts [4,5,6,7]. Given the specifics of these processes, such structural information is particularly important, especially in solution.

In this regard, modern experimental high-resolution NMR methods are among the most informative. 2D correlation experiments designed to transfer coherence through various mechanisms (indirect and direct spin–spin interactions) in many cases allow one to obtain comprehensive information about the chemical structure (topology of a molecule) within even a single method [8].

However, situations sometimes arise where purely experimental approaches do not provide information on the structure, and additional NMR methods are required [9,10]. In such a situation, one can return to analyzing NMR spectral parameters, but at a different level. Namely, these parameters strongly depend on the electronic distribution in the molecule, and their analysis can help in establishing the structure. For example, quantum chemical calculations of chemical shifts for ^1^H, ^13^C, ^15^N, and ^31^P atoms are successfully used both for organic molecules [11,12,13,14,15,16,17,18], and in more difficult cases, for transition metal complexes [19,20,21,22,23]. In this regard, the spin–spin coupling constants (SSCC) look promising, especially in the analysis of the three-dimensional structure.

In terms of NMR theory, there should be a certain relationship between the SSCCs and the mutual orientation of the interacting nuclei. Some empirical correlations between SSCCs and structure have been known for a long time (for example, the Karplus-type dependence [24]) and have been successfully used to solve stereochemical problems [25,26]. In this regard, the use of SSCCs between heavier nuclei looks promising, since both their signs and absolute values can be very informative. However, there may be no empirical dependencies for such SSCCs. Therefore, the natural continuation of our work on the structure of organophosphorus compounds is an attempt to learn how to evaluate a fine NMR parameter—SSCCs at the phosphorus atom.

SSCCs are extremely important in the structural studies of molecules (e.g., determining configuration, conformation, tautomeric structure, and the nature of the chemical bond). However, this is a much more difficult task, since SSCCs are second-order molecular properties and are determined by a linear-response function that accounts for all excited states. Therefore, for example, it is believed that for the calculation of SSCC it is necessary to consider electron correlation, at least at the second-order perturbation theory level, to use special basic sets, to take into account solvent effects, vibrational corrections, and relativistic effects in the case of heavy nuclei [27,28,29]. All this requires substantial computational resources and specialized software products that are not widely available, making such methods practically inapplicable for relatively large molecules of practical interest.

At the same time, there are attempts to calculate the ^1^H-^1^H and ^13^C-^13^C SSCCs in certain systems within the framework of the density functional theory (DFT) [30,31,32,33,34,35,36]. In some cases, the authors were able to obtain calculated values with practically reliable accuracy.

Given that in practice we often deal with phosphorus-containing compounds, estimating spin–spin coupling constants involving a phosphorus atom is becoming particularly relevant. There are only a few examples of calculations of ^31^P-^13^C and ^31^P-^31^P SSCC [37,38,39,40]. Therefore, as a first step in this direction, we will try to consider the scopes and limitations in calculating ^1^*J*_PP_ in the framework of DFT in organophosphorus systems.

## 2. Result and Discussion

To assess the scopes and limitations of theoretical calculations, almost all known types of structures, including P-P bonds for which experimental coupling constants ^1^*J*_PP_ are available, were used as models (Figures S1–S4). This set represents a wide range of possible classes of organophosphorus compounds, with ^1^*J*_PP_ values from −700 to 800 Hz (Table S1). To find an approximation applicable to relatively large molecules, we started our analysis within the DFT framework using a relatively simple combination (PBE0/6-31G(d)//PBE0/6-31G(d) (the “level 1//level 2” notation means: “level 1” at spin–spin constant calculation stage and “level 2” at the geometry optimization stage)) that has proven itself well in the analysis of chemical shifts in organic systems.

**Figure 1:**
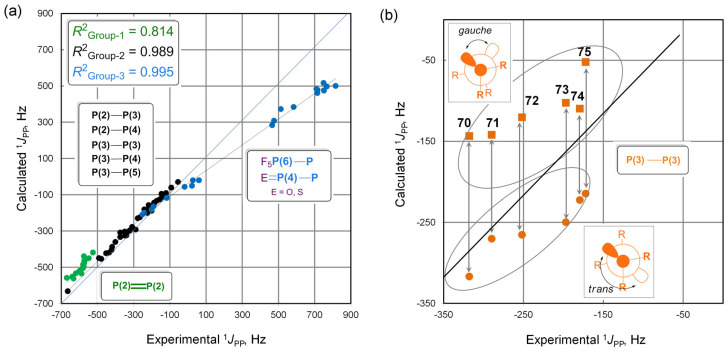
Correlation of calculated (PBE0/6-31G(d)//PBE0/6-31G(d)) vs. experimental ^1^*J*_PP_ for model compounds, with the exception of systems with conformational exchange (**a**) and for systems in conformational exchange (**b**).

According to calculations, a certain correlation is observed between the calculated and the experimental data (Figure 1a). Importantly, in the vast majority of cases, both the absolute values of the constants and their signs are reproduced quite well. In a number of cases, when the original source lacked information about the sign of the experimental ^1^*J*_PP_, its sign in the analysis was taken in accordance with the calculation (Table S1).

In general, three correlations can be clearly identified (Figure 1a, green, black, and blue circles). There are also several points that deviate noticeably from these correlations (Figure 1b, orange circles) (**70**–**75**). These points correspond to diphosphanes of the R_1_R_2_P–PR_1_R_2_ type. It will be shown later that there is no clear conformational preference for them, and they are most likely in a conformational exchange.

The first pronounced correlation (Figure 1a, green, Group 1) with large negative values of ^1^*J*_PP_ (−670 to −526 Hz) is due to systems in which phosphorus atoms participate in a double bond (compounds with coordination numbers of P(2)). The correlation line has a slope of about 1, but the calculation overestimates the experimental values by about 155 Hz for all compounds of this type.

The second correlation that stands out extends from about the middle of the ranges to extremely large positive values of ^1^*J*_PP_ (from −250 to 815 Hz, Figure 1a, blue, Group 3). This group includes systems containing at least one phosphorus atom with coordination numbers of P(4) or P(6), and are characterized by the presence of strongly electronegative atoms directly bonded to it. These are mono and doubly oxidized (sulfidized) diphosphine derivatives (R_2_(E)PPR_2_, R_2_(E)PP(E)R_2_, E=O, S), H_n_Me_3−n_PPF_5_, and cationic bis-chelate complex **61**. For these systems, according to calculations (in vacuum), the forms with an *anti*-orientation of the P=E (E=O, S) groups are significantly more stable than *gauche*- (Table S2); therefore, only ^1^*J*_PP_ for this conformer was considered in the analysis. The correlation line for them deviates strongly from the diagonal (slope of approximately 0.7). For these systems, there is a noticeable underestimation of the ^1^*J*_PP_, which becomes larger as the absolute (positive) values increase.

The remaining data fall on the third correlation in the central part (Figure 1a, black, Group 2), and the ^1^*J*_PP_ covers a wide range from −665 to −55 Hz. Compounds in this group are characterized by single bonds between the phosphorus atoms, which have coordination numbers 2, 3, 4, and 5. The slope of the correlation line is close to 1, and only a slight overestimation (at about 36 Hz) is observed throughout the range.

Thus, there are three fairly good correlations between the calculated (PBE0/6-31G(d)//PBE0/6-31G(d)) and experimental ^1^*J*_PP_. However, they differ depending on the type of P-P fragment. This may be due to limitations of the approximation (simplicity of the basis set, type of functional, etc.), which can lead to systematic errors in calculations that differ for different types of systems. In an attempt to obtain a unified correlation, we tested a number of computational approximations.

**Figure 2:**
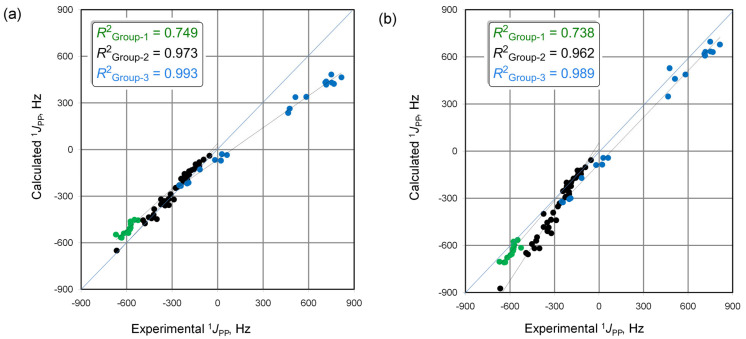
Correlation of calculated vs. experimental ^1^*J*_PP_ for all model compounds (except for systems with conformational exchange): PBE0/6-31+G(d)//PBE0/6-31+G(d) (**a**) and PBE0/6-311G(2d,2p)//PBE0/6-31+G(d) (**b**) levels (the designations are the same as in Figure 1).

First, we tried to use a more flexible basis set with the addition of a diffuse function both at the geometry optimization stage and when calculating the NMR parameters (PBE0/6-31G+(d)//PBE0/6-31G+(d)). In this case, the slopes of the correlation lines are approximately the same as in the initial protocol. However, unexpectedly, the correlation coefficients decrease somewhat for each group (Figure 2a, Table S1).

Next, a combination was examined that proved to be effective for NMR ^31^P shifts [18], in which the shielding is calculated using a more flexible triple-ζ quality basis set (PBE0/6-311G(2d,2p)//PBE0/6-31+G(d)). In this case, three separate correlation lines are also observed, but their slopes differ noticeably from those in the initial approach (Figure 2b). Significant changes are observed for the central (Group 2, slope 1.4 vs. 1.0) and right (Group 3, slope 1.0 vs. 0.7) correlation lines, while there are practically no changes for the left one (Group 1, slope 1.1 vs. 1.1) (Figure 2b, Table S1). It should also be noted that for each of the correlation lines, the *R*^2^ decreases, although the opposite was expected.

**Figure 3:**
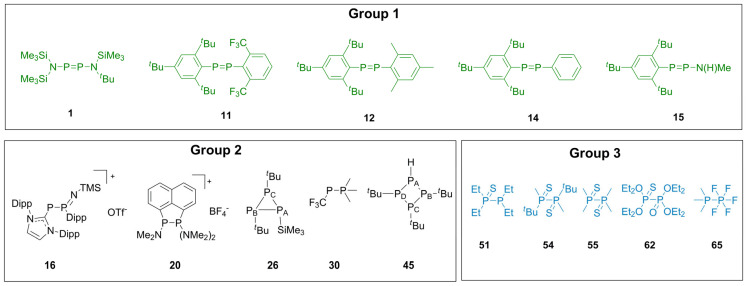
“Training” set of model compounds.

Since a simple approach based on the use of more flexible basis sets did not lead to the desirable unification of correlation dependencies, and, moreover, revealed unexpected effects, a more detailed analysis of the influence of parameters on the calculation results was carried out on a narrower representative “training” set of 15 compounds (18 data points, **1**, **11**–**12**, **14**–**16**, **20**, **26**, **30**, **45**, **51**, **54**–**55**, **62**, and **65**, Figure 3), for which 3 dependencies are also observed, as for the entire set of model compounds (Figure S5).

**Figure 4:**
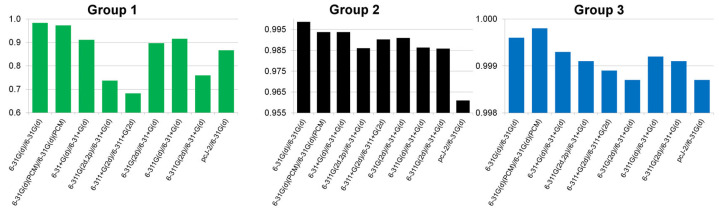
*R*^2^ dependence of on basis set for three groups of the “training” set (the PBE0 functional was used in all cases).

First of all, the possible influence of the solvent was evaluated within the framework of the polarizable continuum model (PCM) [41] (PBE0/6-31G(d)(PCM)//PBE0/6-31G(d)(PCM)). For this purpose, a fairly polar acetone was used as a model solvent in the calculations. In this case, the correlation worsens for Group 1 and Group 2, while almost no changes are expected for Group 3 compared with the initial level (Figure 4, Table S3).

At the same time, the use of a more flexible basis set at both stages of the calculation (PBE0/6-311+G(2d)//PBE0/6-311+G(2d), Table S3) does not lead to an improvement in the correlation, although the slopes become approximately the same as for the PBE0/6-311G(2d,2p)//PBE0/6-31+G(d) level.

In order to establish at which stage (optimization or calculation of constants) the effect of changing the slope of the correlation lines appears when using a more flexible basis set, calculations were carried out with a step-by-step strengthening of the basis set at the SSCC calculation while maintaining optimization at the PBE0/6-31+G(d) level. The evaluation showed that in the series of basis sets 6-31(2d), 6-311(d), and 6-311(2d), it is the transition to the triple-ζ quality basis set that leads to a significant change in the slopes of all correlation lines, although they remain separate, as in the initial approximation (Figure S6).

Using a specially developed basis set for the SSCC calculations with an increased flexibility in the core region also does not lead to significant changes. Namely, when using the pc*J*-2 basis set obtained by adding tight *s*, *p*, *d,* and *f* functions to the standard polarization-consistent basis sets pc-*n* [42], a picture is very similar to that observed when using PBE0/6-311G(2d,2p) (Table S3). That is, first of all, the effects of triple splitting of the pc*J*-2 basis set are activated. The correlation coefficients are also worse than for the initial approximation for all three groups of compounds (Figure 4).

**Figure 5:**
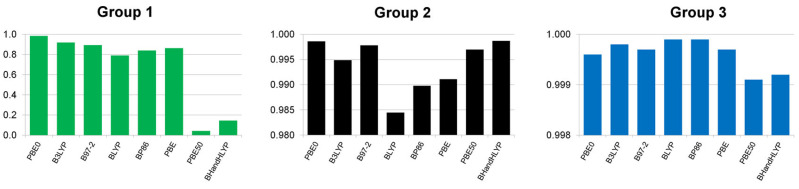
*R*^2^ dependence on the functionals when calculating the SSCCs ^1^*J*_PP_ for three groups of the “training” set (in all cases, the 6-31G(d) basis set and the PBE0/6-31G(d)) geometry was used).

The type of functional can also influence the calculated NMR parameters. So, when using other standard hybrid functionals (B3LYP, B97-2), the changes for Group 2 and Group 3 are not minor compared to the original approach (Figure 5, Table S3). In Group 1 (with P=P), *R*^2^ is significantly lower (0.919 and 0.893 vs. 0.984; Figure 5, Table S3).

Pure-GGA functionals (BLYP, BP86, PBE), which are often recommended in the literature (e.g., KT-3 [43]), were also tested. For Group 3, virtually no changes were observed, for Group 2 the correlation worsened somewhat, but for Group 1 (with P=P), a noticeable deterioration in the correlation was noted compared to the initial level (0.790, 0.841 and 0.864 vs. 0.984, Figure 5, Table S3).

**Figure 6:**
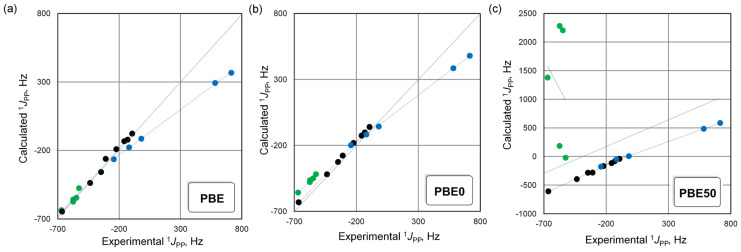
Correlation of calculated vs. experimental ^1^*J*_PP_ for the “training” set, depending on the type of functional: PBE (**a**), PBE0 (**b**), and PBE50 (**c**) (in all cases the 6-31G(d) basis set and the PBE0/6-31G(d)) geometry was used, the designations are the same as in Figure 1).

Next, the influence of more subtle parameters of the functional on the ^1^*J*_PP_ calculation was considered. In particular the dependence of the ^1^*J*_PP_ on the percentage of the HF exact exchange contribution to the functional was estimated. Calculations were performed using the PBE functional with a systematic increase in the proportion of the HF exact exchange contribution: PBE (0%), PBE0 (25%) and PBE50 (50%) (Table 1). Thus, for the systems in the Group 1, with an increase of the HF contribution, the slope of the correlation line increases sharply (Figure 6) and for PBE50, the ^1^*J*_PP_ takes on extreme values (up to 2280 Hz, Table 1). For the Group 3, the slope of the correlation line becomes closer to 1. For the Group 2, the slope decreases only slightly (0.98, Figure 6). Thus, for systems with a P=P fragment, an extremely strong dependence on the proportion of HF exact exchange contribution is observed.

The observed effect indicates the exceptional importance of the correct choice of a functional for the SSCCs calculations. In this context, it is interesting to evaluate another popular functional with the same 50% HF contribution—BHandHLYP. This also leads to a similar “error” for systems with the P=P fragment (Table 1), while for the other two groups, no significant changes are observed (Figure 5, Table S3). Thus, even the proportion of the HF exact exchange contribution to the hybrid functional may, in some cases, lead to unrealistic results.

This “error” may be related to the so-called triplet instability discovered in HF calculations of ^1^*J*_PP_ [44]. The following arguments go some way to suggesting that these breakdowns in accuracy may be related to this. First, the specific ΔE^TS^ dependence: in the classical case, the lowest triplet state should be below the singlet ground state [44]. In our case, although the triplet state is not lower than the ground state, ΔE^TS^ systematically decreases with the increase in the proportion of the HF exact exchange contribution for the compounds of the Group 1 (e.g., for **11** ΔE^TS^ is about 30.0 (PBE), 26.8 (PBE0), 23.5 (PBE50) kcal/mol), but for compounds from the Groups 2 and 3 the opposite picture is observed (Table S4). Second, specific effects on *J*^SD/FC^ terms, involving triplet perturbation operators [45]. In our case, with the increase in the HF contribution, the SD and FC components for compounds of Group 1 suffer the most (Table S5). Moreover, the magnitude of the “error” correlates with the percentage of the HF exact exchange contribution.

A clear explanation of the identified anomalies requires a separate detailed analysis, which may become the subject of further research. However, based on the main objective of this work—to find a tool for practical assessments—it is possible to recommend the use of hybrid functionals in which the proportion of the HF exact exchange contribution is significantly less than 50%.

### 2.1. Practical Aspects and Recommendations

Testing a number of standard approaches in the calculation of the ^1^*J*_PP_ within the framework of the DFT method did not allow us to identify a unified correlation for all of the above-mentioned systems. Moreover, unexpected effects related to the flexibility of the basis sets and the contribution of exact HF exchange to the functional were discovered, examples of which had not previously been observed for ^1^*J*_PP_. Thus, we concluded that, for now, the question of finding a protocol to unify correlation dependencies remains open. At the same time, the obtained results allow us to estimate the ^1^*J*_PP_ with good accuracy within a certain series of compounds. From a practical point of view, the dependencies obtained indicate the possibility of using reliable protocols, but with an appropriate correction specific to each group.

If we analyze the revealed correlation dependencies in more detail, it becomes clear that for calculating the ^1^*J*_PP_, the best agreement with the experiment is achieved at the PBE0/6-31G(d)//PBE0/6-31G(d) level (Table 2). At the same time, across different types of systems, there are three correlation lines, each with a systematic error. To obtain a good quantitative estimate of the ^1^*J*_PP_, such errors can be minimized by performing a linear correction procedure [46,47] according to Equation (1) using regression analysis parameters for the corresponding type of model systems:

*J*^corrected^ = (*J* − **Intercept**)/**Slope** (1)

where *J* is the calculated ^1^*J*_PP_, and **intercept** and **slope** are scaling factors.

**Figure 7:**
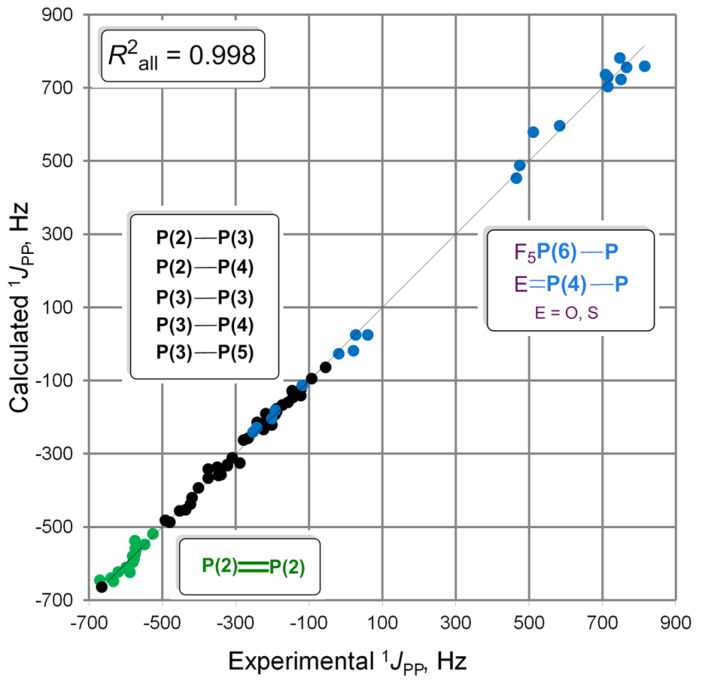
Correlation of calculated (corrected, PBE0/6-31G(d)//PBE0/6-31G(d) level) vs. experimental ^1^*J*_PP_ for all model compounds (the designations are the same as in Figure 1).

Thus, the application of this procedure allows one to obtain ^1^*J*_PP_, which can be quantitatively compared with experimental data (Figure 7). It can be seen that the best accuracy is achieved for compounds in the central part (Group 2), for which the error is minimal (*RMSE* = 13 Hz). For P=P type compounds (Group 1), the accuracy is somewhat lower (*RMSE* = 17 Hz). The lowest accuracy is expected for compounds from Group 3 (*RMSE* = 27 Hz) (Table 2).

### 2.2. Systems with Conformational Exchange

As shown above, for diphosphanes of the R_1_R_2_P–PR_1_R_2_ type (**70**–**75**), the calculated ^1^*J*_PP_ values are in poor agreement with the correlation in the central region. According to calculations for these compounds, the conformers resulting from rotation around the P-P bond are close in energy (Table S6), and as a result, the NMR parameters in solution can be exchange-averaged. The calculated values for these rotamers differ by up to 150 Hz, while experimental values for ^1^*J*_PP_ lie in between. Considering the complexity of theoretically estimating the population of the corresponding forms, comparison of experimental ^1^*J*_PP_ values with those calculated for one conformation may be incorrect, which explains the observed deviation.

**Figure 8:**
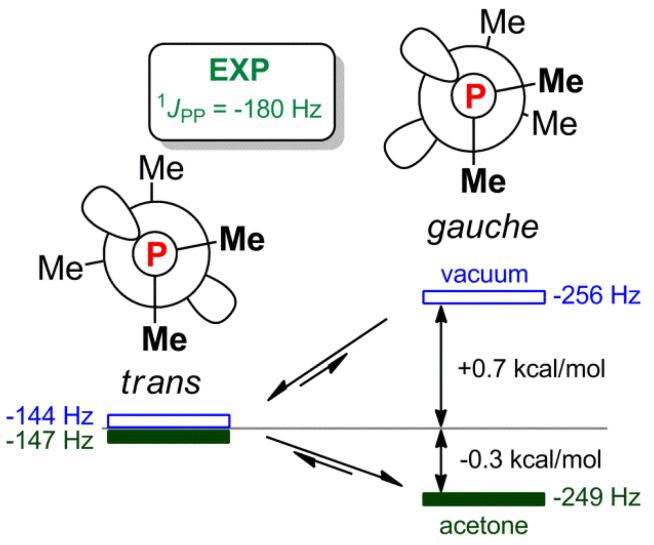
Schematical representation of energy diagrams for *trans* and *gauche* isomers of compound **74** in vacuum and acetone, and corresponding calculated (PBE0/6-31G(d)//PBE0/6-31G(d)) and experimental ^1^*J*_PP_.

For example, for compound **74**, according to calculations, the ^1^*J*_PP_ is −109.6 and −222.4 Hz for *trans* and *gauche* forms, respectively (Figure 8). In this case, the energy difference is small, and the equilibrium can be easily changed by the solvent. Therefore, even a small shift in equilibrium can significantly change the observed ^1^*J*_PP_. Thus, in such systems, the limitations in estimating the ^1^*J*_PP_ are not due to the weakness of the theoretical approximation used to calculate ^1^*J*_PP_, but to the difficulty in predicting the population of forms in solution.

### 2.3. Showcasing Examples

**Figure 9:**
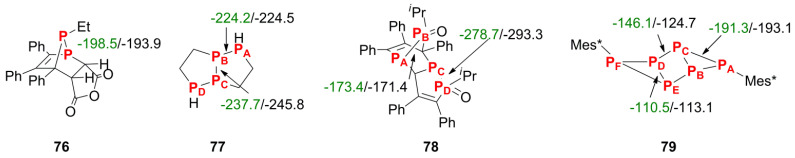
Structure of compounds **76**–**79** with experimental (green) and calculated (black, corrected, PBE0/6-31G(d)//PBE0/6-31G(d)) ^1^*J*_PP_ (Hz) (Mes* = 2,4,6-Tri-tert-butylphenyl).

Having established the optimal protocol for evaluating the ^1^*J*_PP_ on a wide range of different model compounds, we will now attempt to apply it to calculate the ^1^*J*_PP_ for more complex bi- and tri-cyclic strained structures (Figure 9).

For example, for compounds **76** and **77** with two and four phosphorus atoms, which belong to Group 2, the agreement between the ^1^*J*_PP_ calculated according to the proposed protocol and the experimental one is excellent (Figure 9, Table S7). Good agreement is also observed for the tricyclic structure **78**, which belongs to Group 3 (Figure 9, Table S7). For tricyclic hexaphosphane (Mes*P_6_Mes*, **79**, Group 2), the agreement for the two constants is excellent (<2.6 Hz), while for ^1^*J*(P_C_-P_D_) the agreement is somewhat poorer (Figure 9, Table S7). Overall, despite the rather complex picture of the connections in these cyclic systems, the calculation allows us to predict the ^1^*J*_PP_ with good accuracy and in a fairly wide range.

**Figure 10:**
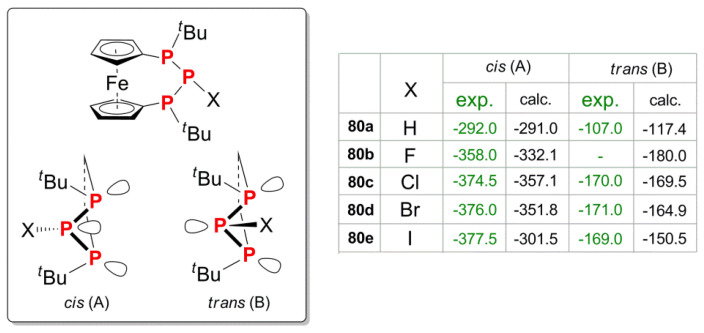
Structure of the triphospha[3]ferrocenophanes (**80a**–**e**) and schematic of the possible diastereomers with the experimental and calculated ^1^*J*_PP_ (Hz, PBE0/6-31G(d)//PBE0/6-31G(d), corrected).

From this perspective, it is interesting to see how this protocol will allow the ^1^*J*_PP_ to be predicted when more subtle structural features such as the isomeric structure are changed. In this regard, the example of triphospha[3]ferrocenophanes (**80a**–**e**) [48] is indicative; in solution, they exist in both *cis* and *trans* forms, and their ratio depends on the substituent on the phosphorus atom (Figure 10). Overall, the results are in fairly good agreement with the experiment (Figure 10), although in some cases the differences are somewhat larger than for relatively simple model compounds. It is important that the trend in ^1^*J*_PP_ is reproduced unambiguously when hydrogen at the phosphorus atom is replaced by halogen in both isomeric forms (Figure 10). However, as we move to heavier halogens, the discrepancy between the calculation and experiment increases (up to 76 Hz for I). This is most likely due to the relativistic effects of a heavy I atom on the NMR parameters of neighboring nuclei [49,50], and in this case, a fully relativistic level of theory may be required. But the most important fact is that the calculations predict a significant difference in the ^1^*J*_PP_ between the isomeric forms (350–360 Hz vs. 165–170 Hz), which is fully consistent with the experiment. Thus, the proposed approach can be reliably used in practice to identify the isomeric structure of phosphorus-containing systems.

## 3. Experimental Section

### Calculational Details

The quantum chemical calculations have been carried out within the framework of the density functional theory (DFT) [51] with the Gaussian 03 [52] (Revision B.04) and the Gaussian 16 [53] (Revision A.03) software packages by using using a variety of functionals (PBE0 [54], PBE [55], PBE50 [56], B3LYP [57,58], B97-2 [59], BLYP [60,61,62], BP86 [60,63], BHandHLYP [64]) and a number of families of basis sets (Pople’s [63,64,65,66,67,68,69,70,71,72], Jensen’s [42]). To take into account the medium effects, calculations were also carried out in the framework of Polarizable Continuum Model [41] (denoted as “PCM”) with acetone as solvent. Wherever possible, geometry optimization was started from X-ray structure. For most of the compounds the calculations were carried out for all possible conformers/isomers and results for the lowest energy forms were used in analysis.

The SSCC was calculated in the framework of the GIAO method [73].

The pcJ-2 basis set was downloaded from the EMSL basis set library for the Gaussian package [74,75,76]. The Gaussian 03 calculations were carried out on a PC with an Intel Core i7-3970X CPU, 3.5 GHz. Calculations with the pc*J*-2 basis set were carried out with the Gaussian 16 on 20 CPUs, Intel Xeon ES-2650 2.20 GHz.

## 4. Conclusions

Thus, it is possible to estimate indirect SSCCs ^1^*J*_PP_ fairly accurately using even modest levels of theory. Certain difficulties may arise only for diphosphanes of the R_1_R_2_P–PR_1_R_2_ type that in solution are in fast (in NMR time scale) exchange of conformers with close populations.

In practice, a relatively simple PBE0/6-31G(d)//PBE0/6-31G(d) combination is sufficient for calculating the SSCCs ^1^*J*_PP_. To obtain estimates with practically reliable accuracy in order to reduce systematic errors, it is necessary to carry out a linear correction procedure specific to different groups of compounds.

The effectiveness of the proposed protocol is demonstrated by the example of SSCCs ^1^*J*_PP_ estimates for relatively complicated systems, as well as for the analysis of finer structural features—the isomeric structure.

The proposed approach allowed the absolute sign of ^1^*J*_PP_ to be determined in a number of cases where it is unknown experimentally.

## Figures and Tables

**Table 1:** Experimental and calculated ^1^*J*_PP_ for the “training” set (basis set in the calculation of SSCCs is 6-31G(d), geometry optimization is the same for all levels—PBE0/6-31G(d)).

Comp.	Exp.	Calculated ^1^*J*_PP_, Hz			
		PBE0	PBE	PBE50	BHandHLYP
**1**	−670.0	−558.1	−635.9	1377.5	529.9
**11**	−574.3	−478.4	−557.9	185.0	17.8
**12**	−573.7	−462.9	−573.6	2279.9	837.3
**14**	−548.7	−450.4	−545.8	2205.2	734.6
**15**	−526.0	−418.1	−475.8	−21.6	−416.2
**16**	−665.0	−631.4	−646.4	−608.8	−643.7
**20**	−436.4	−419.1	−437.0	−394.6	−418.6
**26**	−347.0	−325.2	−357.4	−285.4	−305.2
**30**	−309.5	−276.9	−261.6	−280.9	−277.7
	−222.4	−181.6	−190.9	−167.5	−175.5
	−157.5	−125.3	−132.7	−114.9	−123.4
**45**	−131.1	−102.5	−122.0	−81.7	−90.8
	−93.0	−60.6	−77.6	−42.5	−51.9
**51**	−243.0	−198.9	−264.3	−177.8	−187.4
**54**	−118.0	−117.3	−177.2	−51.6	−64.0
**55**	−18.7	−56.5	−114.8	6.2	−2.6
**62**	583.0	385.0	292.4	483.6	499.4
**65**	715.0	479.2	367.0	586.4	625.2
*R*^2^ (Group 1)		0.9837	0.8638	0.0430	0.1459
*R*^2^ (Group 2)		0.9986	0.9911	0.9970	0.9987
*R*^2^ (Group 3)		0.9996	0.9997	0.9991	0.9992

The experimental values are shown in green, and the largest “errors” are shown in red.

**Table 2:** Empirical scaling factors obtained by the linear regression analysis of the calculated and experimental ^1^*J*_PP_ for corresponding groups of models, *R*^2^ and *RMSE*.

Level of Theory		Intercept	Slope	*R* ^2^	*RMSE ^a^*
PBE0/6-31G(d)// PBE0/6-31G(d)	Group 1	155.0	1.10	0.814	17.0
	Group 2	34.5	1.00	0.989	13.1
	Group 3	−37.1	0.71	0.995	27.1
PBE0/6-31+G(d)// PBE0/6-31+G(d)	Group 1	18.5	0.89	0.749	20.5
	Group 2	40.2	1.07	0.974	20.9
	Group 3	−64.3	0.69	0.994	31.1
PBE0/6-311G(2d,2p)// PBE0/6-31+G(d)	Group 1	14.7	1.10	0.739	21.1
	Group 2	53.6	1.46	0.962	25.2
	Group 3	−79.9	0.99	0.990	40.5

*^a^ RMSE*—Root Mean Square Error (Hz).

## Data Availability

Supplementary Information available. Structures of all model compounds with experimental ^1^*J*_PP_, all calculation results, and some comments.

## Supplementary Materials

The following supporting information can be downloaded at: https://www.mdpi.com/article/10.3390/molecules31111831/s1, Figure S1: The structure of model compounds from Group-1 (**1**–**15**); Figure S2: The structure of model compounds from Group-2 (**16**–**49**); Figure S3: The structure of model compounds from Group-3 (**50**–**69**) (DCHA=dicyclohexylamine). Figure S4: The structure of model compounds in conformational exchange (**70**–**75**); Table S1: Experimental and calculated ^1^*J*_PP_ (Hz) for all model compounds except for systems with conformational exchange (**1**–**69 [77,78,79,80,81,82,83,84,85,86,87,88,89,90,91,92,93,94,95,96,97,98,99,100,101,102,103,104,105,106,107,108,109,110,111,112,113,114,115,116,117,118,119,120,121,122,123,124,125]**); Table S2: Experimental and calculated ^1^*J*_PP_ (Hz) for different forms (with corresponded energy difference ΔE) of the model compounds **50**–**69**; Figure S5: Correlation of calculated (PBE0/6-31G(d)//PBE0/6-31G(d)) vs. experimental ^1^*J*_PP_ for (a) all model compounds and (b) the “training” set (except for systems with conformational exchange); Table S3: Experimental and calculated ^1^*J*_PP_ (Hz) for model compounds of the “training” set (**1**, **11**–**12**, **14**–**16**, **20**, **26**, **30**, **45**, **51**, **54**–**55**, **62**, **65**). Figure S6: Correlation of calculated vs. experimental ^1^*J*_PP_ for the “training” set compounds: PBE0/6-31G(2d)//PBE0/6-31+G(d) (a), PBE0/6-311G(d)//PBE0/6-31+G(d) (b) and PBE0/6-311G(2d)//PBE0/6-31+G(d) (c) levels; Table S4: Energy differences between triplet and singlet states (ΔE_TS_, kcal/mol) for some model compounds from three groups (**2**, **11**, **14**, **20**, **45**, **51**, **62**, **65**); Table S5: Experimental and calculated contribution to FC, SD and PSO components of the ^1^*J*_PP_ (Hz) for some model compounds from three groups (**2**, **11**, **14**, **20**, **45**, **51**, **62**, **65**); Table S6: Experimental and calculated ^1^*J*_PP_ (Hz) for gauche and trans forms (with corresponded energy difference ΔE) of the model compounds **70**–**75** [126,127,128]; Table S7: Experimental and calculated ^1^*J*_PP_ (Hz) for compounds **76**–**79 [129,130]**.
