# Supplementary material for: Combined DFT Protocol for the Calculation of One-Bond 31P-31P Indirect Nuclear Spin–Spin Couplings
Source: Molecules. 2026 May 26;31(11):1831. doi: 10.3390/molecules31111831 (PMC13258075; doi:10.3390/molecules31111831)
Supplement: Supplementary file 1 [file molecules-31-01831-s001.zip › molecules-4274636-supplementary.pdf]

Supplementary Information for:

Combined DFT protocol for the calculation of one-bond ^{31}P-^{31}P Indirect Nuclear Spin-Spin Couplings

*Svetlana A. Kondrashova, Shamil K. Latypov**

Arbuzov Institute of Organic and Physical Chemistry, FRC Kazan Scientific Center of RAS,
Kazan, Tatarstan 420088, Russian Federation

TABLE OF CONTENTS **pages**

Figure S1. The structure of model compounds from Group-1 (**1-15**). 2

Figure S2. The structure of model compounds from Group-2 (**16-49**). 3

Figure S3. The structure of model compounds from Group-3 (**50-69**) (*DCHA*=dicyclohexylamine). 4

Figure S4. The structure of model compounds in conformational exchange (**70-75**). 4

Table S1. Experimental and calculated $^1J_{PP}$ (Hz) for all model compounds except for systems with conformational exchange (**1-69**). 5

Table S2. Experimental and calculated $^1J_{PP}$ (Hz) for different forms (with corresponded energy difference ΔE) of the model compounds **50-69**. 9

Figure S5. Correlation of calculated (PBE0/6-31G(d)//PBE0/6-31G(d)) vs. experimental $^1J_{PP}$ for (a) all model compounds and (b) the "training" set (except for systems with conformational exchange). 10

Table S3. Experimental and calculated $^1J_{PP}$ (Hz) for model compounds of the "training" set (**1, 11-12, 14-16, 20, 26, 30, 45, 51, 54-55, 62, 65**). 11

Figure S6. Correlation of calculated vs. experimental $^1J_{PP}$ for the "training" set compounds: PBE0/6-31G(2d)//PBE0/6-31+G(d) (a), PBE0/6-311G(d)//PBE0/6-31+G(d) (b) and PBE0/6-311G(2d)//PBE0/6-31+G(d) (c) levels. 12

Table S4. Energy differences between triplet and singlet states (ΔE^{TSa}, kcal/mol) for some model compounds from three groups (**2, 11, 14, 20, 45, 51, 62, 65**). 13

Table S5. Experimental and calculated contribution to FC, SD and PSO components of the $^1J_{PP}$ (Hz) for some model compounds from three groups (**2, 11, 14, 20, 45, 51, 62, 65**). 14

Table S6. Experimental and calculated $^1J_{PP}$ (Hz) for *gauche* and *trans* forms (with corresponded energy difference ΔE) of the model compounds **70-75**. 15

Table S7. Experimental and calculated $^1J_{PP}$ (Hz) for compounds **76-79**. 16

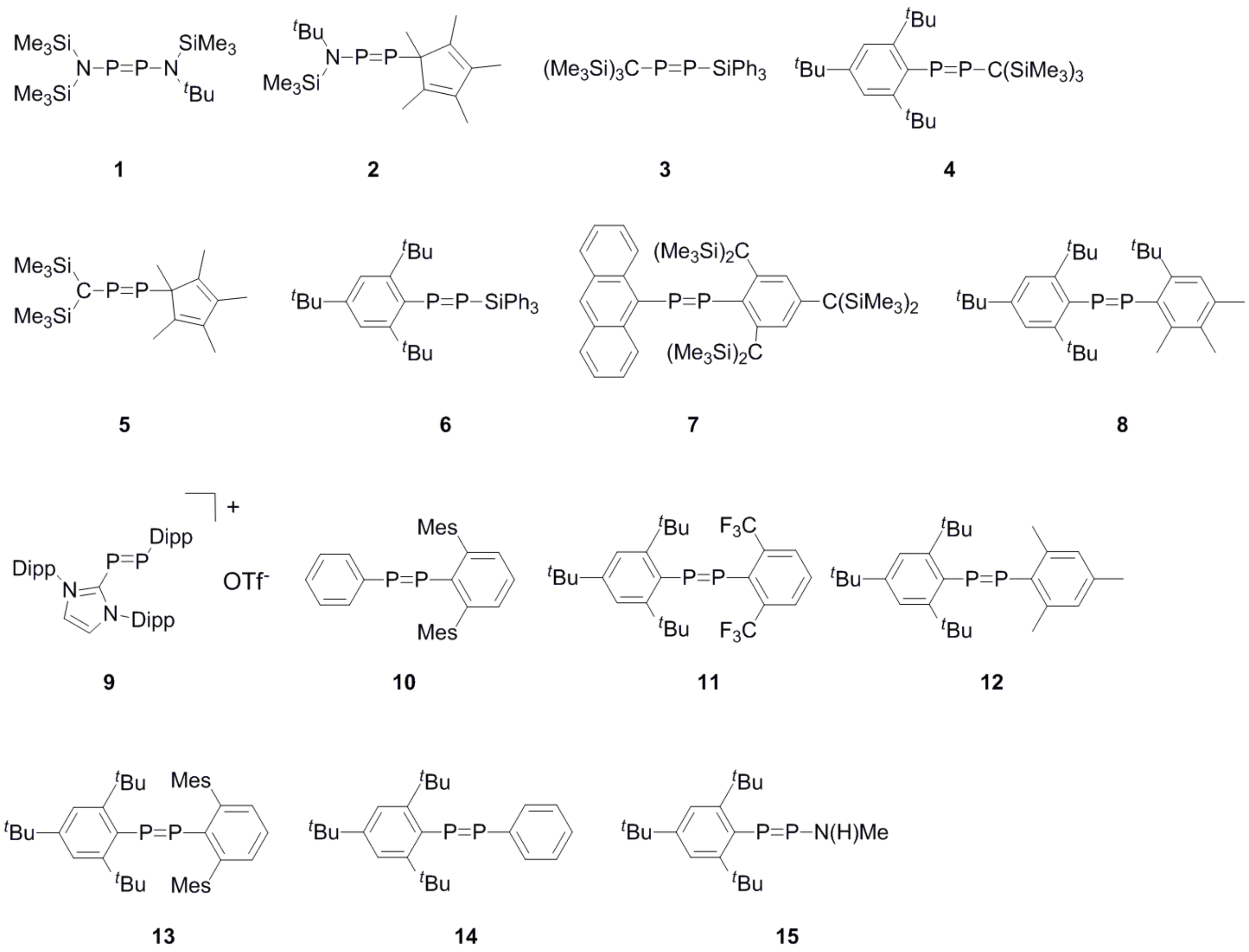

Figure S1. The structure of model compounds from Group-1 (**1-15**).

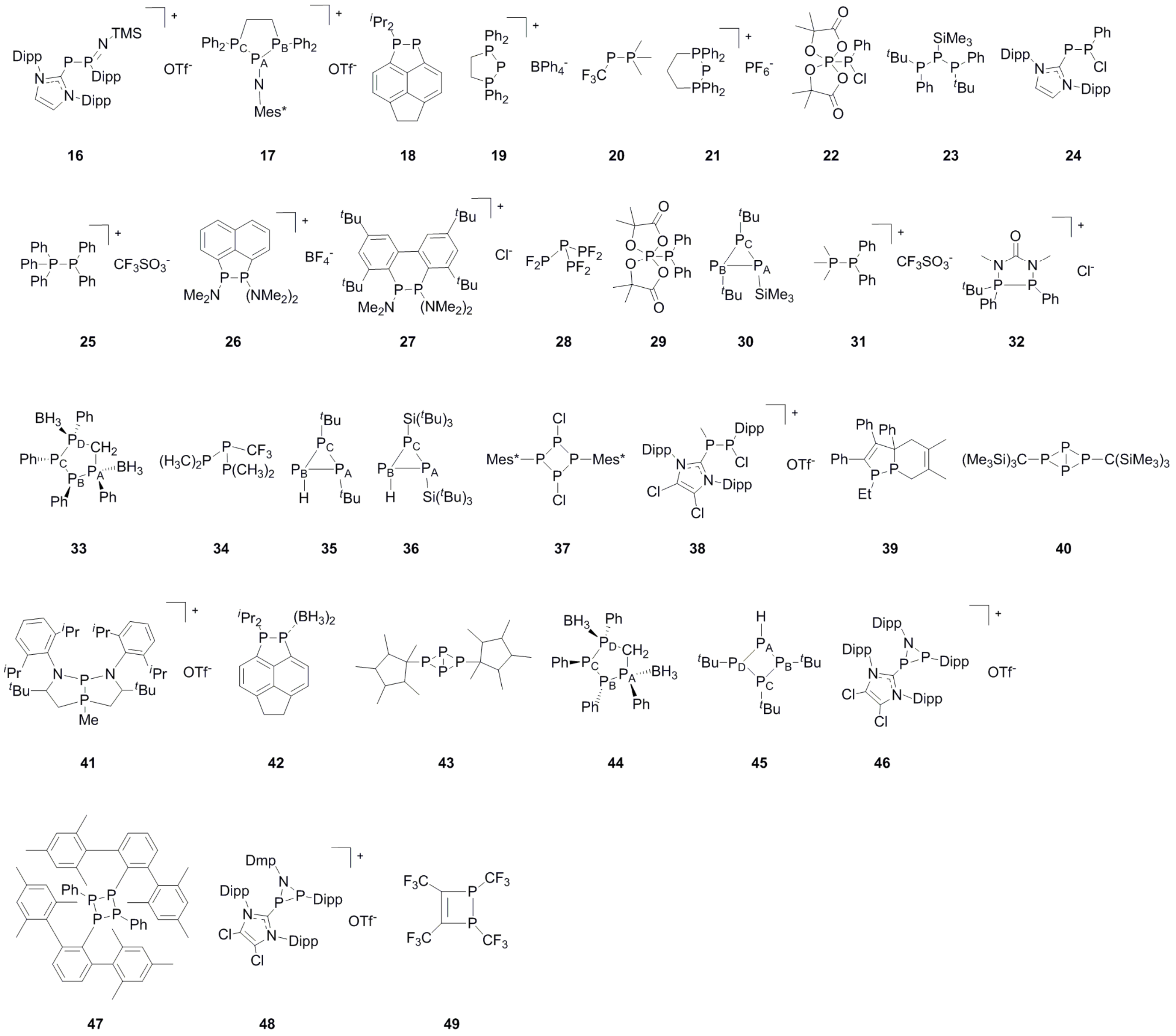

Figure S2. The structure of model compounds from Group-2 (**16-49**).

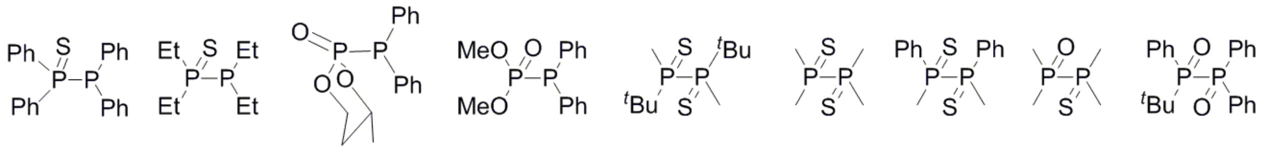

50 51 52 53 54 55 56 57 58

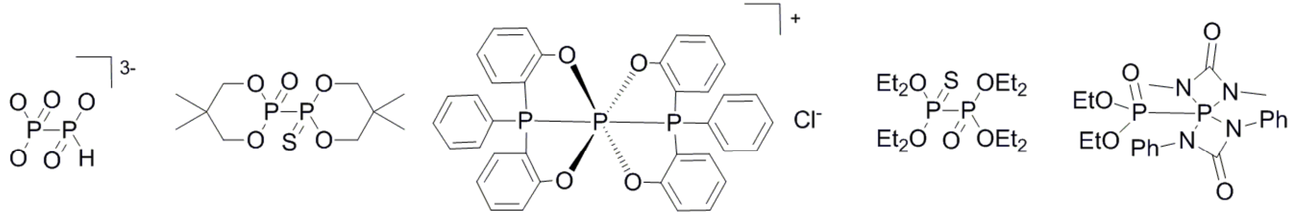

59 60 61 62 63

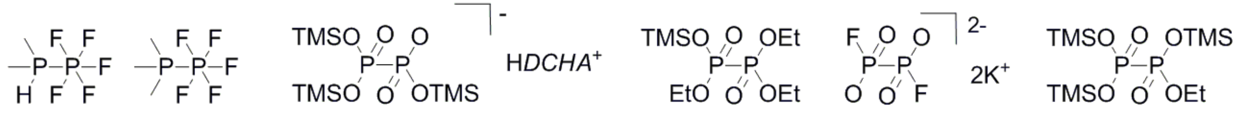

64 65 66 67 68 69

Figure S3. The structure of model compounds from Group-3 (**50-69**) (*DCHA*=dicyclohexylamine).

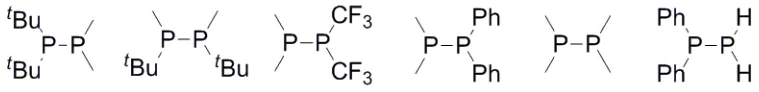

70 71 72 73 74 75

Figure S4. The structure of model compounds in conformational exchange (**70-75**).

Table S1. Experimental and calculated $^1J_{PP}$ (Hz) for all model compounds except for systems with conformational exchange (**1-69**).

**Compound**	Experimental values signs as in the original source[a]	Sign corrected	Experimental values with signs used in this work	Calculated				Reference
				PBE0/6-31G(d)// PBE0/6-31G(d)	PBE0/6-31G(d)// PBE0/6-31G(d), *corrected*	PBE0/6-31+G(d)// PBE0/6-31+G(d)	PBE0/6-311G(2d,2p)// PBE0/6-31+G(d)	
**1**	670	✔	-670.0	-558.1	-645.8	-546.0	-703.1	78
**2**	640	✔	-640.0	-551.1	-639.5	-564.0	-707.7	79
**3**	633.1	✔	-633.1	-562.0	-649.4	-567.0	-706.5	80
**4**	619.7	✔	-619.7	-532.6	-622.7	-539.0	-678.4	81
**5**	599	✔	-599.0	-519.2	-610.6	-532.0	-660.9	79
**6**	588.7	✔	-588.7	-533.8	-623.8	-536.0	-654.6	80
**7**	581	✔	-581.0	-485.4	-580.0	-507.0	-624.2	82
**8**	580	✔	-580.0	-503.7	-596.6	-514.0	-633.4	83
**9**	-577		-577.0	-494.4	-588.1	-511.0	-622.7	84
**10**	575	✔	-575.0	-438.8	-537.8	-463.0	-574.1	85
**11**	574.3	✔	-574.3	-478.4	-573.6	-501.0	-607.7	86
**12**	573.7	✔	-573.7	-462.9	-559.6	-479.0	-594.5	87
**13**	572	✔	-572.0	-467.3	-563.6	-479.0	-595.6	85
**14**	548.7	✔	-548.7	-450.4	-548.3	-452.0	-565.6	87
**15**	526	✔	-526.0	-418.1	-519.0	-454.0	-613.7	88
**16**	-665		-665.0	-631.4	-664.7	-649.0	-873.4	84

**17**	492	✔	-492.0	-448.7	-482.3	-454.0	-648.3	89
			-419.0	-386.4	-420.2	-382.0	-546.8	
**18**	479.6	✔	-479.6	-453.1	-486.7	-475.0	-656.5	90
**19**	453	✔	-453.0	-422.4	-456.1	-434.0	-589.8	91
**20**	-436.4		-436.4	-419.1	-452.8	-442.0	-617.6	92
**21**	423	✔	-423.0	-404.3	-438.0	-419.0	-569.9	91
**22**	401.7	✔	-401.7	-359.8	-393.6	-446.6	-617.5	93
**23**	-374.7		-374.7	-333.0	-366.9	-352.3	-481.7	94
**24**	374.5	✔	-374.5	-308.0	-341.9	-319.0	-397.7	95
**25**	350	✔	-350.0	-302.7	-336.6	-332.0	-453.0	96
**26**	347	✔	-347.0	-325.2	-359.1	-359.5	-508.7	97
**27**	340	✔	-340.0	-323.4	-357.3	-345.0	-486.2	97
**28**	323	✔	-323.0	-296.1	-330.0	-318.2	-437.0	98
**29**	322.2	✔	-322.2	-298.7	-332.6	-357.5	-521.3	93
**30**	-309.5		-309.5	-276.9	-310.9	-286.0	-391.4	99
	-222.4		-222.4	-181.6	-215.7	-182.0	-239.6	
	-157.5		-157.5	-125.3	-159.5	-125.3	-168.5	
**31**	289	✔	-289.0	-291.8	-325.7	-320.0	-438.8	100
**32**	278	✔	-278.0	-229.1	-263.1	-247.0	-351.5	101
**33**	-266.6		-266.6	-223.9	-258.0	-241.1	-332.6	102
	-172.6		-172.6	-133.2	-167.4	-130.8	-173.2	
**34**	242	✔	-242.0	-180.0	-214.1	-187.0	-254.5	103
**35**	-226.6		-226.6	-197.7	-231.8	-206.0	-293.8	99
	-223.7		-223.7	-186.6	-220.7	-185.0	-242.6	
	-144.1		-144.1	-111.9	-146.2	-112.0	-151.6	
**36**	-224.2		-224.2	-199.7	-233.8	-203.0	-289.1	99
	-188.0		-188.0	-142.7	-176.9	-140.0	-196.5	
	-141.3		-141.3	-105.3	-139.6	-105.0	-147.3	
**37**	-218		-218.0	-183.6	-217.7	-164.0	-222.7	104
**38**	-217.8		-217.8	-156.2	-190.4	-155.0	-200.5	105

**39**	210.4	✔	-210.4	-174.6	-208.7	-185.0	-250.6	106
**40**	203.2	✔	-203.2	-161.3	-195.5	-160.0	-219.7	80
**41**	203.1	✔	-203.1	-165.5	-199.7	-187.0	-259.3	107
**42**	201.2	✔	-201.2	-187.7	-221.8	-196.0	-273.8	90
**43**	192	✔	-192.0	-158.4	-192.6	-156.0	-215.2	108
**44**	-188.5		-188.5	-150.3	-184.5	-161.6	-221.6	102
	-146.5		-146.5	-93.9	-128.2	-93.6	-122.0	
**45**	-131.1		-131.1	-102.5	-136.8	-103.0	-149.0	99
	-93.0		-93.0	-60.6	-94.9	-65.0	-101.4	
**46**	-131		-131.0	-105.3	-139.6	-110.0	-149.8	84
**47**	122	✔	-122.0	-90.7	-125.0	-80.0	-120.7	85
**48**	-122		-122.0	-106.7	-141.0	-106.0	-143.6	84
**49**	55	✔	-55.0	-29.3	-63.7	-39.0	-56.0	109
**50**	-252.4		-252.4	-208.1	-241.4	-230.0	-319.8	110
**51**	243	✔	-243.0	-198.9	-228.4	-233.0	-324.5	111
**52**	±202.3	✔	-202.3	-181.3	-203.5	-217.0	-304.6	112
**53**	192	✔	-192.0	-165.3	-181.0	-213.0	-298.3	113
**54**	±118	✔	-118.0	-117.3	-113.2	-128.0	-169.8	114
**55**	±18.7	✔	-18.7	-56.5	-27.4	-65.4	-86.8	115
**56**	±21.0	✔	+21.0	-50.2	-18.5	-71.0	-85.1	116
**57**	27	✔	+27.0	-19.4	25.0	-29.6	-41.7	117
**58**	60.4		+60.4	-19.9	24.3	-34.3	-41.9	118
**59**	+465.5		+465.5	284.0	453.2	236.0	347.9	119
**60**	475		+475.0	308.6	487.9	264.3	528.0	120
**61**	512		+512.0	372.8	578.5	338.6	461.7	121
**62**	583		+583.0	385.0	595.7	339.4	488.7	122
**63**	708.9		+708.9	484.2	735.8	433.1	619.1	123
**64**	+714		+714.0	460.8	702.7	418.9	608.6	124
**65**	+715		+715.0	479.2	728.7	439.2	632.6	124
**66**	748		+748.0	516.8	781.8	484.4	697.9	125

**67**	751		+751.0	474.6	722.2	432.3	635.4	125
**68**	766		+766.0	498.0	755.2	423.0	631.8	126
**69**	815		+815.0	500.4	758.6	466.3	679.4	125
R^2 (Group-1)				**0.814**		**0.749**	**0.739**	
RMSE (Group-1)				**17.0**		**20.5**	**21.1**	
R^2 (Group-2)				**0.989**		**0.974**	**0.962**	
RMSE (Group-2)				**13.1**		**20.9**	**25.2**	
R^2 (Group-3)				**0.995**		**0.994**	**0.990**	
RMSE (Group-3)				**27.1**		**31.1**	**40.5**	

[a] In most papers, only the absolute value without explicit sign or the modulus value is given for $^1J_{PP}$. (±)

Table S2. Experimental and calculated ${}^{1}J_{PP}$ (Hz) for different forms (with corresponded energy difference ΔE) of the model compounds **50-69**.

Compound[a]		Experiment	Calculated	
			PBE0/6-31G(d)//PBE0/6-31G(d)	ΔE, kcal/mol
**51**	LP-S *trans*	-243.0	-198.9	0.0
	LP-S *gauche*		-223.4	1.9
**53**	LP-O *trans*	-192.0	-165.3	0.0
	LP-O *gauche*		-228.3	1.5
**54**	S-S *trans*	-118.0	-117.3	0.0
	S-S *gauche*		-151.1	7.2
**55**	S-S *trans*	-18.7	-56.5	0.0
	S-S *gauche*		-67.9	5.2
**56**	S-S *trans*	+21.0	-50.2	0.0
	S-S *gauche*		-53.3	3.5
**60**	O-S *gauche*	+475.0	415.5	2.1
	O-S *trans*		308.6	0.0
**62**	O-S *gauche*	+583.0	385.0	0.0
	O-S *trans*		483.3	2.2
**66**	O-OTMS *cis*	+748.0	352.0	0.0
	O-OTMS *gauche*		338.1	4.6
	O-OTMS *trans*		382.4	2.7
**67**	O-O *gauche*	+751.0	474.6	0.0
	O-O *trans*		458.7	0.9
**68**	F-F *gauche*	+766.0	498.0	0.0
	F-F *trans*		495.9	1.5
**69**	O-O *gauche*	+815.0	500.4	0.0
	O-O *trans*		558.9	3.9

[a] There is only one energy minimum for the other compounds in this Group.

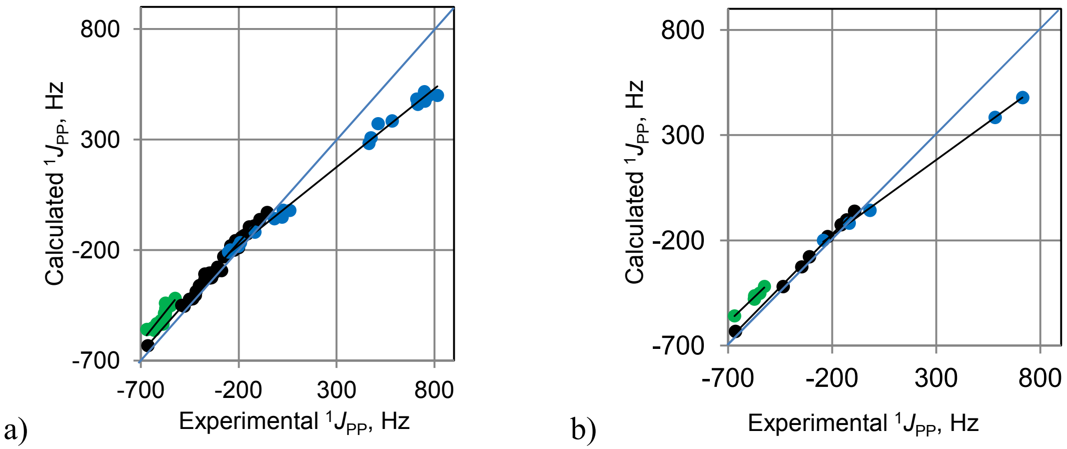

Figure S5. Correlation of calculated (PBE0/6-31G(d)//PBE0/6-31G(d)) vs. experimental $^{1}J_{PP}$ for (a) all model compounds and (b) the "training" set (except for systems with conformational exchange).

Table S3. Experimental and calculated ${}^{1}J_{PP}$ (Hz) for model compounds of the "training" set (**1, 11-12, 14-16, 20, 26, 30, 45, 51, 54-55, 62, 65**).

**Compound**	Experiment	Calculated															
		PBE0/6-31G(d)// PBE0/6-31G(d)	PBE0/6-31G(d)(**PCM**)// PBE0/6-31G(d) (**PCM**)	PBE0/6-31+G(d)// PBE0/6-31+G(d)	PBE0/6-311G(2d,2p)// PBE0/6-31+G(d)	PBE0/6-311+G(2d)// PBE0/6-311+G(2d)	PBE0/6-31G(2d)// PBE0/6-31+G(d)	PBE0/6-311G(d)// PBE0/6-31+G(d)	PBE0/6-311G(2d)// PBE0/6-31+G(d)	PBE0/**pcJ-2**// PBE0/6-31G(d)	**B3LYP**/6-31G(d)// PBE0/6-31G(d)	**B97-2**/6-31G(d)// PBE0/6-31G(d)	**BLYP**/6-31G(d)// PBE0/6-31G(d)	**BP86**/6-31G(d)// PBE0/6-31G(d)	**PBE**/6-31G(d)// PBE0/6-31G(d)	**PBE50**/6-31G(d)// PBE0/6-31G(d)	**BHandHLYP**/6-31G(d)// PBE0/6-31G(d)
**1**	-670.0	-558.1	-539.2	-546.0	-703.1	-712.2	-513.1	-710.7	-703.3	-699.0	-617.1	-519.0	-658.4	-644.1	-635.9	1377.5	529.9
**11**	-574.3	-478.4	-472.4	-501.0	-607.7	-618.1	-456.5	-626.4	-607.6	-593.3	-537.0	-444.1	-582.3	-565.6	-557.9	185.0	17.8
**12**	-573.7	-462.9	-451.7	-479.0	-594.5	-603.6	-438.0	-606.8	-595.1	-569.0	-537.2	-434.6	-597.2	-581.2	-573.6	2279.9	837.3
**14**	-548.7	-450.4	-442.1	-452.0	-565.6	-574.3	-451.9	-574.3	-565.1	-541.9	-510.2	-409.8	-574.9	-559.3	-545.8	2205.2	734.6
**15**	-526.0	-418.1	-416.9	-454.0	-613.7	-629.8	-431.0	-592.1	-610.7	-571.6	-448.4	-340.0	-481.9	-481.2	-475.8	-21.6	-416.2
**16**	-665.0	-631.4	-642.4	-649.0	-873.4	-857.2	-606.1	-901.5	-873.7	-860.6	-667.6	-552.0	-679.6	-660.6	-646.4	-608.8	-643.7
**20**	-436.4	-419.1	-369.9	-442.0	-617.6	-597.5	-428.4	-631.9	-617.6	-436.6	-444.6	-351.1	-460.5	-441.8	-437.0	-394.6	-418.6
**26**	-347.0	-325.2	-322.0	-359.5	-508.7	-488.5	-339.5	-512.8	-509.6	-492.8	-352.6	-289.9	-379.3	-365.8	-357.4	-285.4	-305.2
**30**	-309.5	-276.9	-262.0	-286.0	-391.4	-389.5	-277.2	-387.3	-391.8	-391.1	-270.8	-235.7	-258.0	-262.5	-261.6	-280.9	-277.7
	-222.4	-181.6	-184.6	-182.0	-239.6	-243.1	-171.6	-237.5	-239.4	-240.3	-192.2	-177.2	-199.8	-192.9	-190.9	-167.5	-175.5
	-157.5	-125.3	-132.3	-125.3	-168.5	-164.8	-119.7	-163.3	-167.7	-171.0	-135.7	-128.5	-141.7	-133.8	-132.7	-114.9	-123.4
**45**	-131.1	-102.5	-104.4	-103.0	-149.0	-148.8	-95.1	-148.4	-148.0	-153.6	-117.0	-100.2	-132.5	-126.0	-122.0	-81.7	-90.8
	-93.0	-60.6	-62.1	-65.0	-101.4	-98.8	-61.1	-101.9	-101.0	-103.7	-75.0	-61.0	-88.7	-80.5	-77.6	-42.5	-51.9
**51**	-243.0	-198.9	-226.2	-233.0	-324.5	-320.9	-214.2	-337.0	-325.9	-317.6	-244.2	-193.8	-278.0	-270.0	-264.3	-177.8	-187.4
**54**	-118.0	-117.3	-122.4	-128.0	-169.8	-163.7	-103.9	-186.4	-169.7	-167.6	-145.2	-96.3	-194.3	-183.4	-177.2	-51.6	-64.0
**55**	-18.7	-56.5	-59.3	-65.4	-86.8	-85.7	-44.9	-110.6	-88.4	-83.0	-80.5	-38.5	-128.3	-119.6	-114.8	6.2	-2.6
**62**	+583.0	385.0	398.7	339.4	488.7	504.7	377.1	458.0	489.8	495.2	381.8	405.2	300.4	295.0	292.4	483.6	499.4
**65**	+715.0	479.2	504.8	439.2	632.6	617.0	449.6	583.6	627.8	662.8	486.1	505.3	396.4	377.8	367.0	586.4	625.2
R^2 (Group-1)		**0.9837**	**0.9731**	**0.9109**	**0.7367**	**0.6823**	**0.8970**	**0.9154**	**0.7592**	**0.8662**	**0.9191**	**0.8931**	**0.7904**	**0.8407**	**0.8638**	**0.0430**	**0.1459**
R^2 (Group-2)		**0.9986**	**0.9937**	**0.9937**	**0.9860**	**0.9902**	**0.9909**	**0.9863**	**0.9858**	**0.9609**	**0.9949**	**0.9978**	**0.9845**	**0.9898**	**0.9911**	**0.9970**	**0.9987**
R^2 (Group-3)		**0.9996**	**0.9998**	**0.9993**	**0.9991**	**0.9989**	**0.9987**	**0.9992**	**0.9991**	**0.9987**	**0.9998**	**0.9997**	**0.9999**	**0.9999**	**0.9997**	**0.9991**	**0.9992**

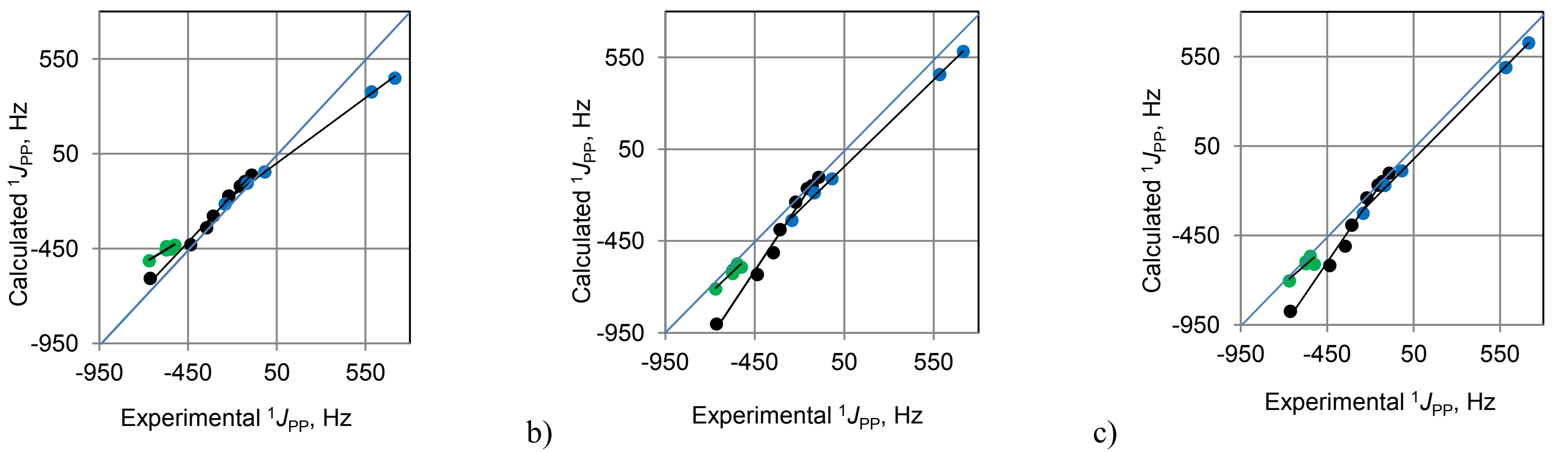

a) b) c)

Figure S6. Correlation of calculated vs. experimental $^{1}J_{PP}$ for the "training" set compounds: PBE0/6-31G(2d)//PBE0/6-31+G(d) (a), PBE0/6-311G(d)//PBE0/6-31+G(d) (b) and PBE0/6-311G(2d)//PBE0/6-31+G(d) (c) levels.

Table S4. Energy differences between triplet and singlet states ($\Delta E^{TS a}$, kcal/mol) for some model compounds from three groups (**2, 11, 14, 20, 45, 51, 62, 65**).

**Compound**		ΔE^{TS}		
		**PBE**/6-31G(d)	**PBE0**/6-31G(d)	**PBE50**/6-31G(d)
Group-1	**2**	27.4	26.1	24.4
	**11**	30.0	26.8	23.5
	**14**	27.3	24.2	20.5
Group-2	**20**	41.4	41.9	42.5
	**45**	44.1	45.4	48.8
Group-3	**51**	48.4	52.0	53.6
	**62**	58.6	72.4	74.5
	**65**	88.7	86.8	92.8

[a] $\Delta E^{TS} = E^{T}-E^{S}$

Table S5. Experimental and calculated contribution to FC, SD and PSO components of the ${}^{1}J_{PP}$ (Hz) for some model compounds from three groups (**2, 11, 14, 20, 45, 51, 62, 65**).

**Compound**		Experiment	Calculated											
			**PBE**/6-31G(d)// PBE0/6-31G(d)				**PBE0**/6-31G(d)// PBE0/6-31G(d)				**PBE50**/6-31G(d)// PBE0/6-31G(d)			
			**FC**	**SD**	**PSO**	**Total**	**FC**	**SD**	**PSO**	**Total**	**FC**	**SD**	**PSO**	**Total**
Group-1	**2**	-640.0	-313	33	-318	-597.0	-283	90	-358	-551.1	-190	423	-395	-160.8
	**11**	-574.3	-248	52	-362	-557.9	-207	128	-400	-478.4	-10	628	-434	185.0
	**14**	-548.7	-247	52	-352	-545.8	-207	147	-391	-450.4	381	2249	-425	2205.2
Group-2	**20**	-436.4	-370	6	-73	-437.0	-352	5	-72	-419.1	-330	4	-70	-394.6
	**45**	-131.1	-180	44	14	-122.0	-166	48	15	-102.5	-149	52	15	-81.7
		-93.0	-145	45	22	-77.6	-133	49	23	-60.6	-118	53	23	-42.5
Group-3	**51**	-243.0	-269	22	-18	-264.3	-206	25	-18	-198.9	-190	27	-15	-177.8
	**62**	+583.0	289	11	-8	292.4	377	14	-7	385.0	472	16	-5	483.6
	**65**	+715.0	358	13	-4	367.0	468	14	-4	479.2	572	17	-3	586.4

Table S6. Experimental and calculated ${}^{1}J_{PP}$ (Hz) for *gauche* and *trans* forms (with corresponded energy difference ΔE) of the model compounds **70-75**.

**Compound**	Experimental values signs as in the original source	Sign corrected	Experimental values with signs used in this work	ΔE, kcal/mol (E^{trans}-E^{gauche})	Calculated[a]		Reference
					LP-LP *gauche*	LP-LP *trans*	
**70**	-318		-318.0	0.8	-350.8	-177.8	127
**71**	-290.0		-290.0	-1.6	-304.2	-176.2	127
**72**	252	✔	-252.0	0.9	-299.3	-154.5	128
**73**	197	✔	-197.0	0.3	-283.8	-136.7	127
**74**	-179.7		-179.7	-0.7	-256.5	-143.9	127
**75**	172.2	✔	-172.2	0.1	-248.6	-86.6	129

[a] at the PBE0/6-31G(d)//PBE0/6-31G(d) level, corrected

Table S7. Experimental and calculated $^{1}J_{PP}$ (Hz) for compounds **76-79**.

**Compound**	Experimental values signs as in the original source	Sign corrected	Experimental values with signs used in this work	Calculated		Reference
				PBE0/6-31G(d)// PBE0/6-31G(d)	PBE0/6-31G(d)// PBE0/6-31G(d), corrected	
**76**	198.5	✔	-198.5	-159.7	-193.9	106
**77**	278.7	✔	-278.7	-244.9	-293.3	130
	173.4	✔	-173.4	-158.5	-171.4	
**78**	∓237.7	✔	-237.7	-211.7	-245.8	131
	∓224.2	✔	-224.2	-190.4	-224.5	
**79**	-191.3		-191.3	-158.9	-193.1	104
	-146.1		-146.1	-90.4	-124.7	
	-110.5		-110.5	-78.8	-113.1	
